# Supplementary material for: Identification and Functional Analysis of Apoptotic Protease Activating Factor-1 (Apaf-1) from Spodoptera litura
Source: Insects. 2021 Jan 13;12(1):64. doi: 10.3390/insects12010064 (PMC7828216; doi:10.3390/insects12010064)
Supplement: Supplementary file 1 [file insects-12-00064-s001.pdf]

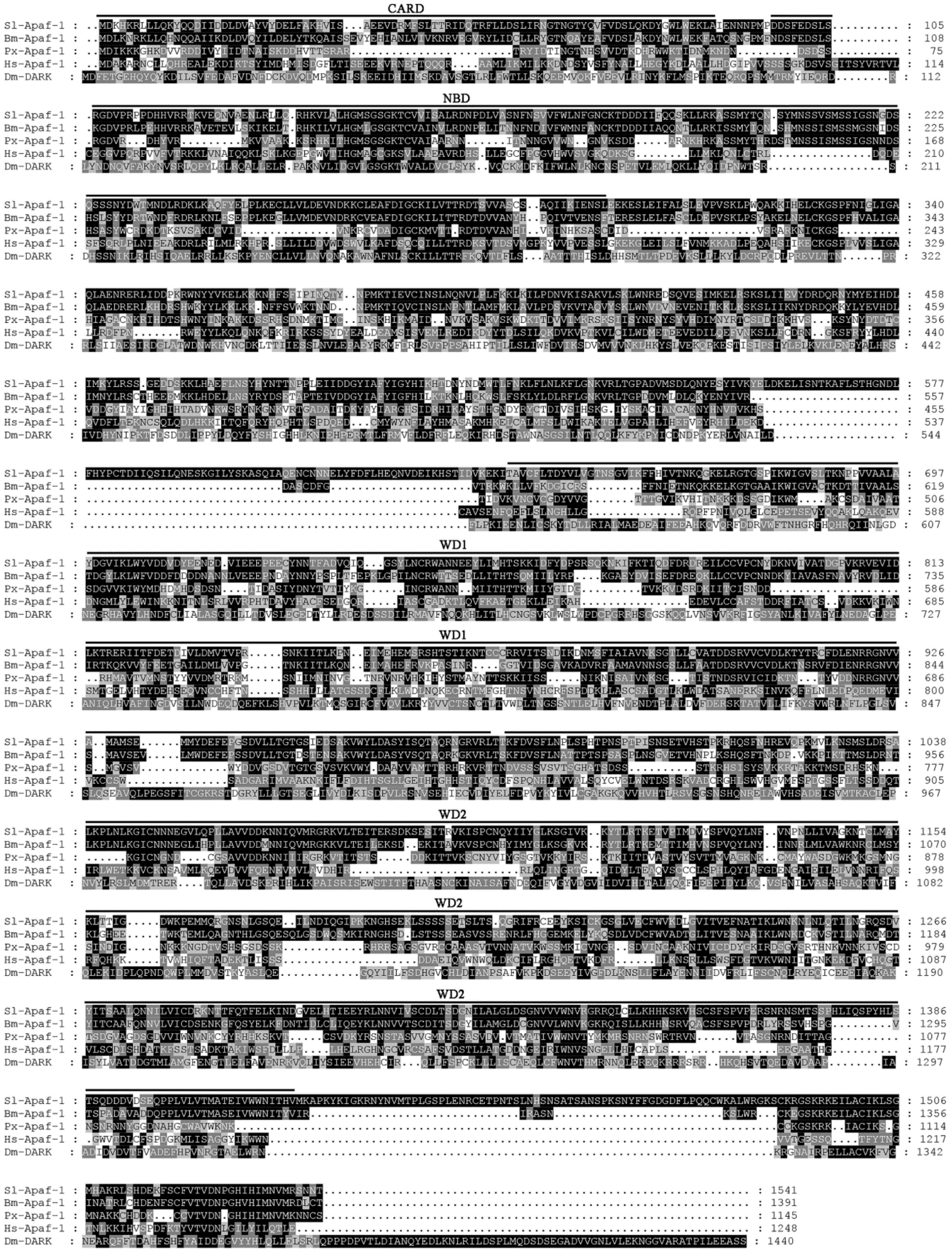

Figure S1. Multiple sequence alignment of Sl-Apaf-1 and other orthologous proteins. The respective positions are indicated on the right. Identical residues between orthologous sequences are shown as white characters against black background, and conservative substitutions are shaded. The CARD, NBD and WD40 domains are highlighted with black lines. GenBank accession numbers: *S. litura* (Sl-Apaf-1, MT793718); *B. mori* (Bm-Apaf-1, XP_012553167); *P. xylostella* (Px-Apaf-1, XP_030028798); *H. sapiens* (Hs-Apaf-1, CAB55588); and *D. melanogaster* (Dm-DARK, AAD45988).

Table S1. Similarity of Sl-Apaf-1 protein with orthologous proteins by BLAST analysis.

Class	Order	Species	Accession	Identities *
Insecta	Lepidoptera	*Spodoptera exigua*	AYM00395	91.97%
		Spodoptera frugiperda	XP_035450484	89.09%
		Helicoverpa armigera	XP_021181657	80.49%
		Trichoplusia ni	XP_026734098	77.61%
		Manduca sexta	XP_030022507	61.44%
		Chilo suppressalis	RVE48846	61.15%
		Bombyx mori	XP_012553167	59.74%
		Pieris rapae	XP_022124006	59.97%
		Plutella xylostella	AHB86312	58.22%
		Danaus plexippus	XP_032516465	53.41%
	Hemiptera	*Acyrthosiphon pisum*	XP_008186216	31.04%
		Aphis glycines	KAE9539441	30.20%
		Aphis gossypii	XP_027847974	30.39%
		Myzus persicae	XP_022163751	30.08%
		Cinara cedri	VVC32818	29.59%
	Coleoptera	*Anoplophora glabripennis*	XP_018564159	28.51%
		Callosobruchus maculatus	VEN55505	28.74%
		Leptinotarsa decemlineata	XP_023029468	28.86%
	Hymenoptera	*Solenopsis invicta*	XP_025993160	28.44%
		Copidosoma floridanum	XP_023246426	29.43%
		Acromyrmex echinatior	XP_011067576	28.30%
		Neodiprion lecontei	XP_015511747	29.91%
	Diptera	*Drosophila melanogaster*	AAD45988	19.00%
Mammalia	Rodentia	*Mus musculus*	AAI31684	21.00%
		Rattus norvegicus	NP_076469	21.00%
	Primates	*Homo sapiens*	CAB55588	20.00%
Osteichthyes	Cypriniformes	*Danio rerio*	NP_571683	21.00%
Amphibia	Anura	*Xenopus laevis*	NP_001085834	20.00%
Nematoda	Rhabditida	*Caenorhabditis elegans*	CAA48781	10.00%

* Amino acid sequence alignment of Sl-Apaf-1 and orthologous proteins from GenBank.

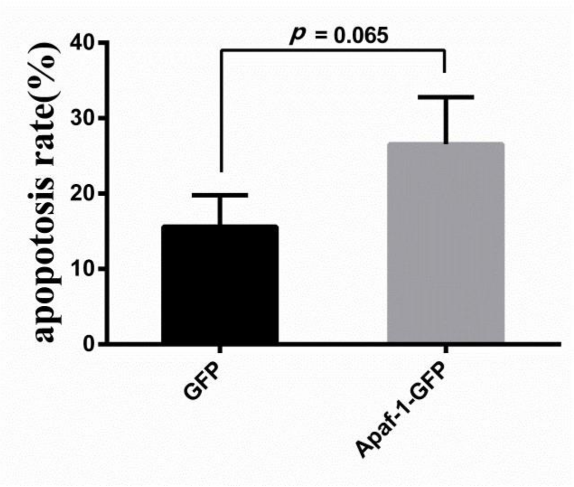

Figure S2. Sl-Apaf-1 overexpression induced apoptosis in SL-1 cell line.

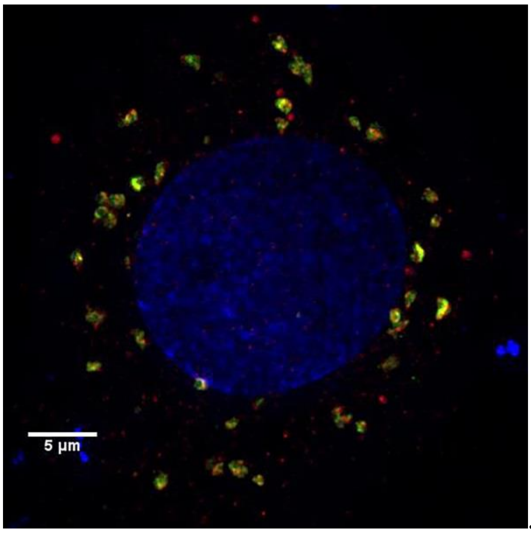

Figure S3. Co-localization of Sl-Apaf-1 and Sl-cytochrome c in a human U2OS cell. Apaf-1 is expressed with EGFP (green) tag and Sl-cytochrome c is expressed with mOrange2 (red) tag. DeltaVision OMX V3 (Marlborough, MA, USA) was used for three-dimensional structured illumination microscopy analysis.

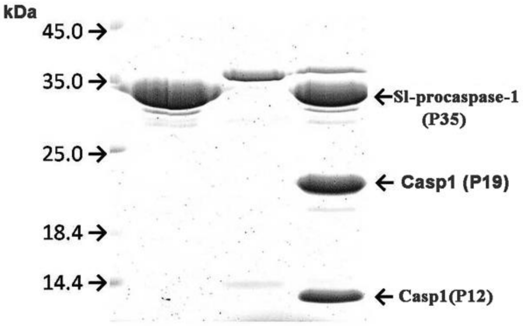

Lane#	1	2	3
Casp1(C178A)	+		+
Casp5		+	+

Figure S4. Sl-caspase-5 directly cleaves Sl-caspase-1. The reaction was performed at 28 °C for 30 min with 2 μM Sl-Caspase-5 recombinant protein and 10 μM Sl-caspase-1(C178A) protein in the reaction buffer (100 mM KCl, 20 mM HEPES, 5 mM DTT, pH 7.5). The reaction was stopped by adding 5× SDS-loading buffer and boiling at 100 °C for 5min. The reaction products were subjected to 15% SDS-PAGE and visualized by Coomassie blue staining. Lane 1: Sl-caspase-1 (C178A); lane2: Sl-caspase-5; lane 3: the reaction products of Sl-caspase-5 and Sl-caspase-1 (C178A).

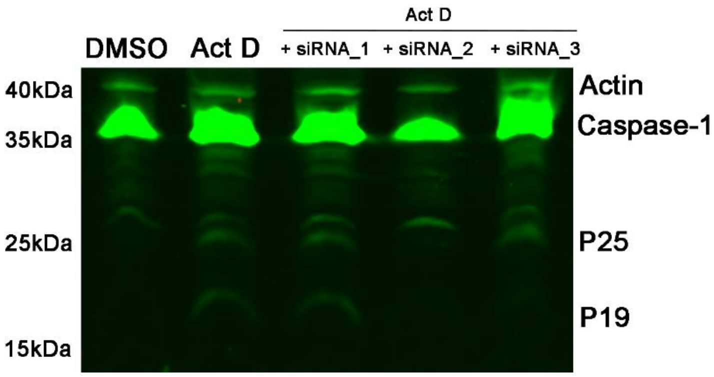

Figure S5. Original western blot figure of Figure 3D (pseudocolor).
